# Supplementary material for: Content-rich biological network constructed by mining PubMed abstracts
Source: BMC Bioinformatics. 2004 Oct 8;5:147. doi: 10.1186/1471-2105-5-147 (PMC528731; doi:10.1186/1471-2105-5-147)
Supplement: Additional File 2 — The original results of the above study (non-essential files are deleted to keep the file size under the limit set by BMC bioinformatics). [file 1471-2105-5-147-S2.bz2 › chilibotAdditionalFile2/dip20/427ID11470772E10357/html/CCL2_CCR2.html]

 


 **CCL2** and **CCR2** 
  
Found 2851 abstracts in PubMed, retrieved 20.  
 

 What does Google say? 
 PDF only 
| .edu only 

---

**Interactive relationship** (e.g. stimulation, inhibition, etc)

**Non-interactive relationship** (e.g. studied together, co-existance, homology, etc.)

- Therefore the current study investigated whether N acetylcysteine NAC, an antioxidative agent, inhibits the interleukin IL 1beta induced expression and production of eotaxin and monocyte chemotactic protein MCP 1  [ **CCR2** ]   [ **CCL2** ]  in human airway smooth muscle cells HASMC.  Ref: 12882449 Eur Respir J, 2003
- MgSO 4 may protect myocardial tissue from ischemic reperfusion injury by inhibiting the expression of Ps of platelets and MCP 1  [ **CCR2** ]   [ **CCL2** ]  mRNA of myocardial tissue and decreasing the concentration of Ca 2 of the serum.  Ref: 12881868 Zhejiang Da Xue Xue Bao Yi Xue Ban, 2003
- Indeed, we find that RP3 alone causes increases in nuclear NF kappaB activity and secretion of MCP 1  [ **CCR2** ]   [ **CCL2** ]  and GM CSF.  Ref: 12881713 Oncogene, 2003
- Furthermore, the inhibitors of mitogen activated protein kinase p38 and extracellular signal regulated kinase 1 2 and nuclear factor kappaB or the activator of peroxisome proliferator activated receptor gamma PPARgamma significantly decreased Hcy induced MCP 1  [ **CCR2** ]   [ **CCL2** ]  and IL 8 secretion in these cells.  Ref: 12881478 Circ ResCirc Res, 2003
- MCP 1  [ **CCR2** ]   [ **CCL2** ]  and IL 8 have been found to express mainly by macrophages in human lesion.  Ref: 12881478 Circ ResCirc Res, 2003
- We detected an upregulation or de novo synthesis of interleukin 1alpha IL 1alpha, IL 1beta, IL 8, macrophage inflammatory protein 1alpha MIP 1alpha, MIP 1beta, GRO alpha, transforming growth factor beta, MCP 1  [ **CCR2** ]   [ **CCL2** ] , IL 2 receptor gamma IL 2Rgamma, IL 10Ralpha, and IL 6R.  Ref: 12874345 Infect Immun, 2003
- IL 6, MIP 2 and MCP 1  [ **CCR2** ]   [ **CCL2** ]  appear to follow the toxicity.  Ref: 12883092 Toxicol SciToxicol Sci, 2003
- These results demonstrate that homocysteine can trigger overexpression of the MCP 1  [ **CCR2** ]   [ **CCL2** ]  gene by altering the intracellular redox status, suggesting that the homocysteine induced changes in the intracellular redox status play an important role in modulating the expression of MCP  [ **CCL2** ]  l in endothelial cells.  Ref: 12883331 J Cardiovasc Pharmacol, 2003
- These data indicate that pathophysiological levels of Hcy can alter human monocyte function by upregulating MCP 1  [ **CCR2** ]   [ **CCL2** ]  and IL 8 expression and secretion via enhanced formation of intracellular ROS originated from NAD P H oxidase source via calmodulin or protein kinase C signaling pathways.  Ref: 12881478 Circ ResCirc Res, 2003
- The protein level of MCP 1  [ **CCR2** ]   [ **CCL2** ]  in the mild hypothermic subgroups was 8.7 7.6 ng.g tissue 1 6 h after reperfusion P <.  Ref: 12887739 Zhonghua Yi Xue Za Zhi, 2003
- The expression of Ps of platelets and CD11b of leukocytes was determined by flowcytometry and the expression of MCP 1  [ **CCR2** ]   [ **CCL2** ]  mRNA of myocardial tissue was measured by RT PCR admission and at 8 60 min of reperfusion R5, R30, R60, R180, R360.  Ref: 12881868 Zhejiang Da Xue Xue Bao Yi Xue Ban, 2003
- At all the concentrations tested, MOM was more effective than DEX in inhibiting ICAM 1 expression and MCP 1  [ **CCR2** ]   [ **CCL2** ]  release p< ; 0.05, each comparison.  Ref: 12877820 Pulm Pharmacol Ther, 2003
- These effects of Hcy were primarily mediated by reactive oxygen species ROS through NAD P H oxidase, because Hcy could upregulate the production of ROS and the inhibitors of protein kinase C, calmodulin, free radical scavengers, or NAD P H oxidase abolished Hcy induced ROS production and MCP 1  [ **CCR2** ]   [ **CCL2** ]  and IL 8 secretion in these cells.  Ref: 12881478 Circ ResCirc Res, 2003
- Angiotensin II induces several fibrogenic chemokines, namely monocyte chemoattractant protein 1  [ **CCL2** ]  MCP 1  [ **CCR2** ]   [ **CCL2** ]  and transforming growth factor beta.  Ref: 12882873 Diabetes Care, 2003
- The aim of the study was to evaluate the inhibitory effect of two corticosteroids, mometasone furoate MOM and dexamethasone DEX, respectively, on a variety of fibroblast functions DNA synthesis and proliferation, expression of adhesion molecules intercellular adhesion molecule 1 ICAM 1, CD54 and hyaluronic cellular adhesion molecule HCAM, CD44 and release of chemokines cytokines monocyte chemoattractant protein MCP 1  [ **CCR2** ]   [ **CCL2** ] , eotaxin, interleukin IL 6 and transforming growth factor TGF beta.  Ref: 12877820 Pulm Pharmacol Ther, 2003
- Monocyte chemoattractant protein 1  [ **CCL2** ]  MCP 1  [ **CCR2** ]   [ **CCL2** ]  and interleukin 8 IL 8 are major chemokines for leukocyte trafficking and have been identified in atheromatous plaques.  Ref: 12881478 Circ ResCirc Res, 2003
- The expression of MCP 1  [ **CCR2** ]   [ **CCL2** ]  positive cells almost coincided with the macrophages.  Ref: 12892039 J Cardiol, 2003
- The protein levels of IL 1beta and MCP 1  [ **CCR2** ]   [ **CCL2** ]  in the cortical ischemic core were measured by ELISA.  Ref: 12887739 Zhonghua Yi Xue Za Zhi, 2003
- In contrast, staining for MCP 1  [ **CCR2** ]   [ **CCL2** ]  was little seen in the stellate vascular smooth muscle cells.  Ref: 12892039 J Cardiol, 2003
- Monocyte chemoattractant protein MCP 1  [ **CCR2** ]   [ **CCL2** ]  and macrophage colony stimulating factor M CSF are two of the associated chemokines, but their role in coronary artery disease has not been sufficiently clarified.  Ref: 12892039 J Cardiol, 2003
- We found that clinically relevant levels of Hcy 10 to 1000 micro mol L increased the protein secretion and mRNA expression as well as activity of MCP 1  [ **CCR2** ]   [ **CCL2** ]  and IL 8 in cultured primary human monocytes.  Ref: 12881478 Circ ResCirc Res, 2003
- Urinary MCP 1  [ **CCR2** ]   [ **CCL2** ]  levels decreased from 0.456 0.22 ng mg creatinine to 0.08 0.096 ng mg creatinine.  Ref: 12882873 Diabetes Care, 2003
- Although no associations were found in alleles or genotypes, MCP 1  [ **CCR2** ]   [ **CCL2** ]  2518A G genotype affected the age at onset of PD patients.  Ref: 12889089 Mov Disord, 2003
- The MCP 1  [ **CCR2** ]   [ **CCL2** ]  protein level in the cortical ischemic cores of the normothermic subgroups began to increase since the 6th hour afer reperfusion 22.5 8.7 ng.g tissue 7 times that in the sham operation samples, P <.  Ref: 12887739 Zhonghua Yi Xue Za Zhi, 2003
- The inhibition of PNF by AsA was associated with decreased intra islet levels of inflammation related molecules IL 1, TNF alpha, iNOS, COX 2 and chemokines MCP 1  [ **CCR2** ]   [ **CCL2** ]  and MIP 3alpha.  Ref: 12879250 DiabetologiaDiabetologia, 2003
- Whereas treatment with infectious virus induced a rapid down regulation of the  **CCL2**  monocyte chemoattractant protein 1  [ **CCL2** ]  MCP 1  [ **CCR2** ]   [ **CCL2** ]  specific receptor  **CCR2** , inactivated virus did not significantly alter  **CCR2**  surface expression.  Ref: 12885942 J Leukoc Biol, 2003
- Because MCP 1  [ **CCR2** ]   [ **CCL2** ]  induces monocyte immigration and differentiation to macrophages, which augment extracellular matrix production and tubulointerstitial fibrosis, pharmacological reduction of angiotensin II may also exert its beneficial effects in diabetic nephropathy by downregulation of renal MCP 1  [ **CCR2** ]   [ **CCL2** ] .  Ref: 12882873 Diabetes Care, 2003
- ... TNF alpha induced a significant upregulation in ICAM 1 expression and in MCP 1  [ **CCR2** ]   [ **CCL2** ] , eotaxin and IL 6 release p< ; 0.05, each comparison.  Ref: 12877820 Pulm Pharmacol Ther, 2003
- CONCLUSION Mild hypothermia reduces the level of MCP 1  [ **CCR2** ]   [ **CCL2** ]  in the cortex after cerebral ischemia reperfusion which may be one of the important mechanisms of the neuroprotective effects of mild hypothermia.  Ref: 12887739 Zhonghua Yi Xue Za Zhi, 2003
- MgSO 4 inhibited the expression of Ps of platelets and MCP 1  [ **CCR2** ]   [ **CCL2** ]  mRNA of myocardial tissue and decreased serum Ca 2 concentration, but did not affect the expression of CD11b of leukocytes.  Ref: 12881868 Zhejiang Da Xue Xue Bao Yi Xue Ban, 2003
- NAC 10 mM decreased the expression of eotaxin and MCP 1  [ **CCR2** ]   [ **CCL2** ] , by 46 11% n=7 and 87 4% n=6, respectively.  Ref: 12882449 Eur Respir J, 2003
- and weak for IL 1beta and monocyte chemotactic peptide 1 MCP 1  [ **CCR2** ]   [ **CCL2** ] .  Ref: 12874304 Infect Immun, 2003
- In parallel, the response to  **CCL2**  MCP 1  [ **CCR2** ]   [ **CCL2** ]  was lost after infection with active virus Neither a  **CCL2**  MCP 1  [ **CCR2** ]   [ **CCL2** ]  induced shift of intracellular calcium concentrations nor the chemotactic response to  **CCL2**  MCP 1  [ **CCR2** ]   [ **CCL2** ]  was detectable.  Ref: 12885942 J Leukoc Biol, 2003
- ... IL 6, MIP 2 and MCP 1  [ **CCR2** ]   [ **CCL2** ]  were increased by 4 8 hours.  Ref: 12883092 Toxicol SciToxicol Sci, 2003
- This study investigated the expression of MCP 1  [ **CCR2** ]   [ **CCL2** ]  and c fms M CSF receptor in human coronary tissues.  Ref: 12892039 J Cardiol, 2003
- OBJECTIVE To investigate the effect of ischemic reperfusion I R on the expression of P selectin Ps of platelets, CD11b of leukocytes, monocyte chemotactic protein 1  [ **CCL2** ]  MCP 1  [ **CCR2** ]   [ **CCL2** ]  mRNA of myocardial tissue and the protective role of MgSO 4.  Ref: 12881868 Zhejiang Da Xue Xue Bao Yi Xue Ban, 2003
- However, the M CSF c fms system, rather than MCP 1  [ **CCR2** ]   [ **CCL2** ] , is more important in the late stage of restenosis.  Ref: 12892039 J Cardiol, 2003
- In a mouse macrophage like cell line, the same beta isoform activation increases the release of the MCP 1  [ **CCR2** ]   [ **CCL2** ]  chemokine.  Ref: 12892998 Mol Aspects MedMol Aspects Med, 2003
- CONCLUSION On the basis of previous studies and the present case, the high production of IL 8 and MCP 1  [ **CCR2** ]   [ **CCL2** ]  by monocytes in LPS stimulated WBCC from periodontitis patients is most likely acquired, as their levels decrease over time when the periodontal infection is controlled.  Ref: 12887346 J Clin PeriodontolJ Clin Periodontol, 2003
- Homocysteine stimulated MCP 1  [ **CCR2** ]   [ **CCL2** ]  mRNA expression and protein production in a time dependent and dose dependent manner in endothelial cells, decreased intracellular glutathione GSH and protein thiol levels, as well as G6PDH activity and NADPH levels.  Ref: 12883331 J Cardiovasc Pharmacol, 2003
- ACE Inhibitors Improve Diabetic Nephropathy Through Suppression of Renal MCP 1  [ **CCR2** ]   [ **CCL2** ] .  Ref: 12882873 Diabetes Care, 2003
- CONCLUSIONS Both MCP 1  [ **CCR2** ]   [ **CCL2** ]  and the M CSF c fms system are involved in the atherogenesis of de novo lesions.  Ref: 12892039 J Cardiol, 2003
- Neutralizing antibodies against human RANTES, MIP 1alpha, MIP 1beta, alpha interferon IFN alpha, IFN beta, IFN gamma, interleukin 4 IL 4, IL 10, IL 13, IL 16, MCP 1  [ **CCR2** ]   [ **CCL2** ] , MCP  [ **CCL2** ]  3, tumor necrosis factor alpha TNF alpha, or TNF beta failed to reverse the HIV 1 suppressive activity.  Ref: 12885891 J VirolJ Virol, 2003
- Up regulation of IL 6, IL 8, and MCP 1  [ **CCR2** ]   [ **CCL2** ]  mRNA expression and production by the virus in combination with opaque S. pneumoniae was two to threefold higher than that induced by the virus combined with the transparent S. pneumoniae variant.  Ref: 12874304 Infect Immun, 2003
- We examined if blockade of the renin angiotensin system lowered urinary levels of the chemokine MCP 1  [ **CCR2** ]   [ **CCL2** ]  and correlated urinary MCP 1  [ **CCR2** ]   [ **CCL2** ]  uMCP 1 with parameters of renal function and glucose and lipid metabolism before and after 1 year of treatment with an ACE inhibitor.  Ref: 12882873 Diabetes Care, 2003
- However, both S. pneumoniae opacity variants increased mRNA expression of MIP 1alpha, MIP 1beta, IL 6, and MCP 1  [ **CCR2** ]   [ **CCL2** ]  in HMEE cells activated by a prior influenza A virus infection compared to levels in cells treated with either agent alone.  Ref: 12874304 Infect Immun, 2003
- The possible connection between periodontitis and atherosclerosis through IL 8 and MCP 1  [ **CCR2** ]   [ **CCL2** ]  is discussed.  Ref: 12887346 J Clin PeriodontolJ Clin Periodontol, 2003
- OBJECTIVE To investigate the effect of intraischemic mild hypothermia on the protein levels of interleukin IL 1beta and monocyte chemoattractant protein MCP 1  [ **CCR2** ]   [ **CCL2** ]  in the ischemic core of rat cortex after transient focal cerebralischemia.  Ref: 12887739 Zhonghua Yi Xue Za Zhi, 2003
- High levels of RANTES CCL5, MCP 1  [ **CCR2** ]   [ **CCL2** ]   **CCL2** , IP 10 CXCL10, and Mig CXCL9 simultaneously with mononuclear cell infiltration in the lungs was found.  Ref: 12875978 Am J Pathol, 2003
- We studied polymorphisms in the genes for monocyte chemoattractant protein 1  [ **CCL2** ]  MCP 1  [ **CCR2** ]   [ **CCL2** ]  and CC chemokine receptor CCR 2  [ **CCR2** ]  in 171 Parkinson s disease PD patients and 340 controls.  Ref: 12889089 Mov Disord, 2003
- the expression of CD11b of leukocytes was significantly higher than that of the control groups P< ; 0.01 and P< ; 0.05 the expression of MCP 1  [ **CCR2** ]   [ **CCL2** ]  mRNA of myocardial tissue began to rise at R60, reaching the highest at R360 P< ; 0.01 the concentration of Ca 2 of the serum was significantly higher than that of the control groups P< ; 0.01 and P< ; 0.05.  Ref: 12881868 Zhejiang Da Xue Xue Bao Yi Xue Ban, 2003
- This effect was also detected in a second PD group, suggesting a possible involvement of MCP 1  [ **CCR2** ]   [ **CCL2** ]  in PD.  Ref: 12889089 Mov Disord, 2003
- The release of the chemokines interleukin IL 8 and macrophage chemoattractant protein MCP 1  [ **CCR2** ]   [ **CCL2** ]  in the cultures decreased twofold over time.  Ref: 12887346 J Clin PeriodontolJ Clin Periodontol, 2003
- ... the MCP 1  [ **CCR2** ]   [ **CCL2** ]  release was decreased by 69 41% n=10.  Ref: 12882449 Eur Respir J, 2003
- The following primary antibodies were used anti MCP 1  [ **CCR2** ]   [ **CCL2** ] , anti c fms, anti macrophages and anti alpha smooth muscle actin.  Ref: 12892039 J Cardiol, 2003
- Expression patterns of MCP 1  [ **CCR2** ]   [ **CCL2** ]  and c fms exhibited no difference between the two lesion types.  Ref: 12892039 J Cardiol, 2003
